# Abrupt shifts in 21st-century plankton communities

**DOI:** 10.1126/sciadv.abf8593

**Published:** 2021-10-29

**Authors:** B. B. Cael, Stephanie Dutkiewicz, Stephanie Henson

**Affiliations:** 1 National Oceanography Centre, Southampton, UK.; 2 Massachusetts Institute of Technology, Cambridge, MA, USA.

## Abstract

Marine microbial communities sustain ocean food webs and mediate global elemental cycles. These communities will change with climate; these changes can be gradual or foreseeable but likely have much more substantial consequences when sudden and unpredictable. In a complex virtual marine microbial ecosystem, we find that climate change–driven shifts over the 21st century are often abrupt, large in amplitude and extent, and unpredictable using standard early warning signals. Phytoplankton with unique resource needs, especially fast-growing species such as diatoms, are more prone to abrupt shifts. Abrupt shifts in biomass, productivity, and community structure are concentrated in Atlantic and Pacific subtropics. Abrupt changes in environmental variables such as temperature and nutrients rarely precede these ecosystem shifts, indicating that rapid community restructuring can occur in response to gradual environmental changes, particularly in nutrient supply rate ratios.

## Teaser

Climate change is projected to cause large, abrupt shifts in subtropical plankton communities.

## INTRODUCTION

Phytoplankton are the foundation of global ocean ecosystems; they are responsible for about half of global primary production (*1*), and they also mediate biogeochemical cycles and thereby regulate climate (*2*). Plankton community structure and function are the emergent result of numerous environmental factors including insolation, nutrient concentrations/supplies, temperature, carbon chemistry, and the fluid motions in which they are embedded (*3*). They are therefore sensitive to environmental forcing. Plankton communities are already changing with climate (*4*) and are expected to continue to do so, possibly with catastrophic consequences (*5*). Plankton community responses to climate change are therefore a critical component of the ecological and socioeconomic consequences of climate change, ranging from biodiversity loss to fisheries sustainability, as well as global climate feedbacks.

Plankton communities are complex systems composed of many organisms competing for resources, grazing on each other, consuming compounds produced by one another, and interacting in various other ways (*6*, *7*). How a community responds to environmental change is therefore nontrivial; the community may be resilient and not change, may change in a gradual/proportional fashion, or may exhibit a rapid and/or large, nonlinear response (*8*). Furthermore, environmental change may itself be gradual or abrupt and may be multifaceted, with multiple stressors occurring simultaneously (*9*). Rapid, nonlinear changes, whether these are rapid community readjustments to gradual environmental change or commensurate responses to rapid environmental change, are intrinsically more difficult to characterize, predict, and plan for. Both for climate policy and ecosystem management, the possibility of environmental changes triggering “tipping point” responses in ocean ecology and biogeochemistry is a substantial concern (*10*). Abrupt shifts in plankton can result in wholesale shifts to an alternative ecosystem state, affecting food webs, elemental cycling, and even fisheries, such as after the North Pacific regime shifts in 1977 and 1989 (*11*).

If and when abrupt shifts in plankton communities do occur, they may be predictable using so-called early warning signals (EWSs). Nonlinear dynamical systems in many cases exhibit certain properties when being forced toward a critical transition, which manifest as both spatial and temporal statistical signals (*12*). Experimental evidence suggests that EWSs sometimes precede abrupt ecosystem shifts in aquatic systems, both in marine (*13*) and freshwater (*14*) environments, and sometimes by more than a decade in the latter case, but not always (*15*). In some cases, EWS may not act as an alarm system for abrupt shifts because of the underlying system dynamics, characteristics of the forcing (such as strong seasonality or the existence of multiple drivers), and characteristics of the data [such as measuring the wrong variables or at insufficient time resolution (*16*)] or may even be false alarms (*17*). Nonetheless, when EWSs do presage abrupt ecological shifts, there is the opportunity to plan for, or even avoid crossing, a critical transition.

Therefore, it is valuable to discern which components or properties of plankton communities are predisposed to sudden shifts, when and where they are likely to occur, and whether early warning statistical signals are capable of predicting them. To this end, we simulated the response of complex virtual plankton communities to a climate change scenario over the 21st century, investigating the presence and predictability of abrupt shifts. The simulation that we present is the most complex marine microbial ecosystem model included in a climate change simulation to date, with 35 phytoplankton (analogs of prokaryotes, picoeukaryotes, coccolithophores, diazotrophs, diatoms, and mixotrophic dinoflagellates) and 16 zooplankton, collectively spanning a 4000-fold size range (fig. S1) (*18*). Results from the simulation of the current-day conditions (figs. S2 and S3) compare well in patterns and magnitudes to observations of phytoplankton size classes, e.g., (*19*), and functional groups (*20*), making this model well suited to exploring plankton functional group dynamics. Similar versions of the model have also been compared extensively against functional groups and size classes of plankton (*18*, *21*, *22*). This virtual ecosystem is perturbed with a high emission scenario [similar to Representative Concentration Pathway 8.5 scenario (*23*)] that results in ∼3°C sea surface temperature warming by 2100, sea ice retreat, increased stratification, and an altered overturning circulation (*24*, *25*). We focus on locations equatorward of 65°N, although range expansions do occur at higher latitudes (fig. S4; Materials and Methods).

**Fig. 1.**
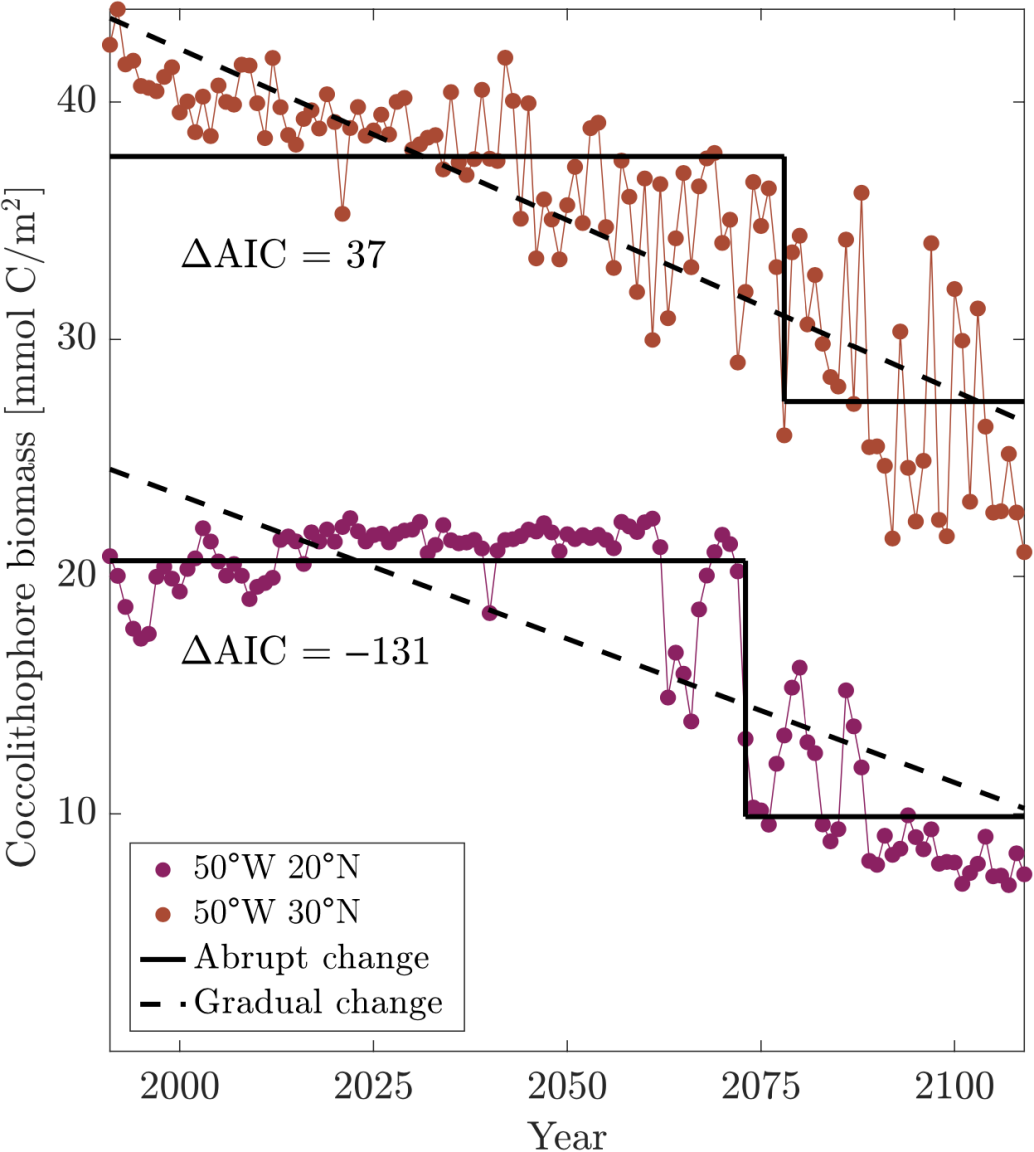
Example time series demonstrating abrupt shift. Annual time series of depth-integrated coccolithophore biomass at two model grid points. Black solid lines show step-function fit and timing of change points; black dashed lines show linear fit. ΔAIC indicates the difference in each fit’s Akaike information criterion value, with positive (negative) ΔAIC meaning that the linear (step-function) model is a better fit. In this case, the time series at 30°N does not exhibit an abrupt change, while the time series at 20°N does.

**Fig. 2.**
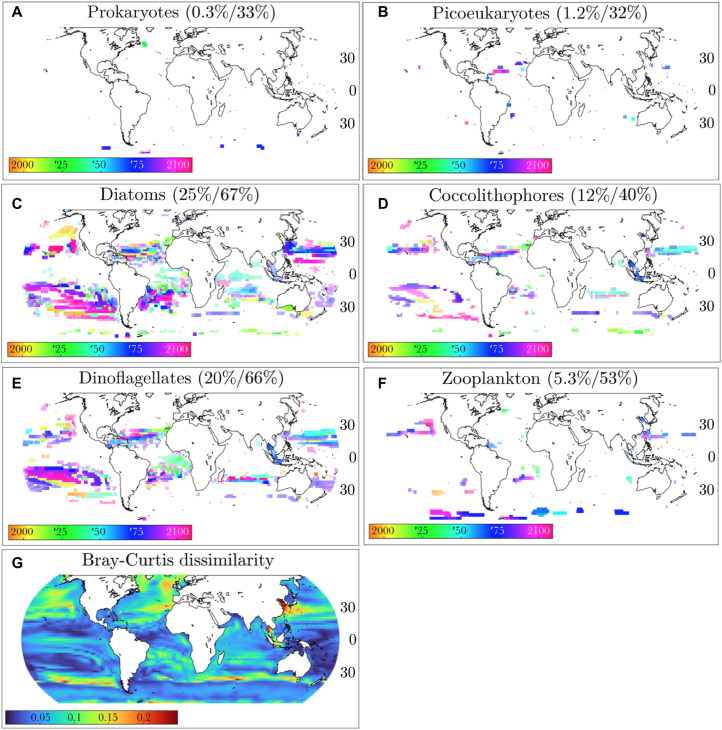
Maps of abrupt shifts for plankton functional types. (**A** to **F**) Pixel color indicates year of abrupt shift; pixel intensity indicates magnitude of shift. Percentages are the fraction of ocean area equatorward of 65° that have abrupt changes >25% (left) and that have either abrupt nonlinear or gradual linear changes >25% (right). (**G**) Bray-Curtis dissimilarity between the phytoplankton communities at each grid point averaged over 1990–2000 versus averaged over 2100–2110.

**Fig. 3.**
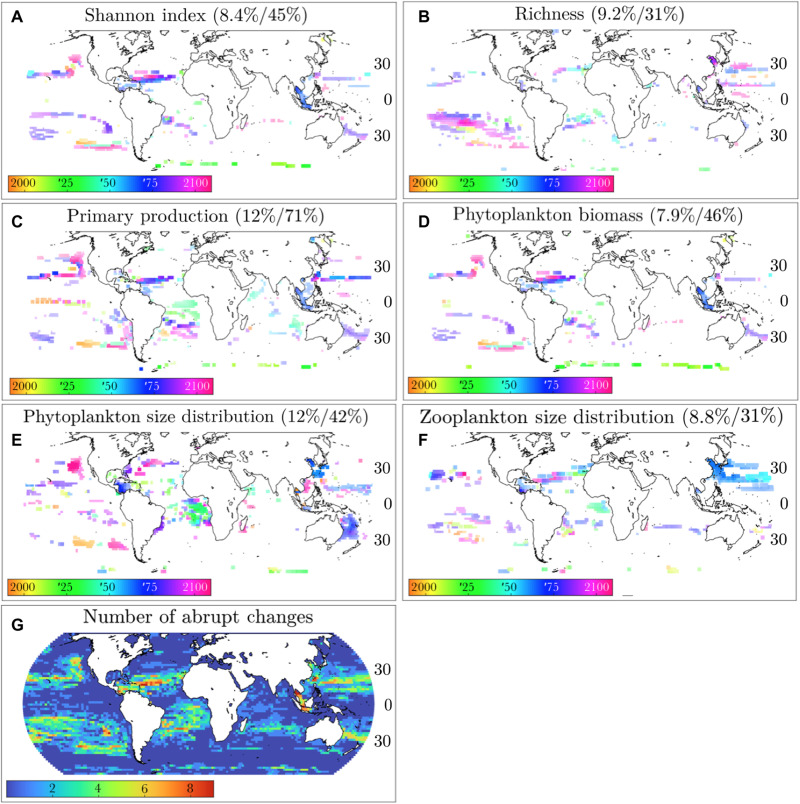
Maps of abrupt shifts for ecosystem properties. (**A** to **F**) Pixel color indicates year of abrupt shift; pixel intensity indicates magnitude of shift. Percentages are the fraction of ocean area equatorward of 65° that have abrupt changes >10% (left) and that have either abrupt or gradual changes >10% (right). (**G**) Number of abrupt shifts detected in all metrics [Fig. 2 (A to F) and (A to F)] for each grid point.

We define abrupt shifts as the change point in a time series that is better fit by a step function than a linear trend (Fig. 1). This generic definition is used to capture changes that are better described as instantaneous rather than gradual. We also require abrupt shifts to be large in magnitude: >25% in the case of phytoplankton functional type biomass and >10% change in other properties. This difference in cutoff value is because individual plankton types’ biomasses are necessarily more variable than whole-ecosystem biomass or other properties. Other cutoff values provided qualitatively similar results (see Materials and Methods). We focus on shifts that are also large in spatial extent, i.e., occur simultaneously in many adjacent grid cells. We focus on single abrupt shifts in any given time series because we are interested in large, persistent, centennial-scale ecological shifts. We analyze the time series of the depth-integrated biomass of each phytoplankton functional type (Fig. 2) and of the following ecosystem properties (Fig. 3): Shannon’s index for phytoplankton, phytoplankton richness, primary production, total phytoplankton biomass, the phytoplankton size distribution, and the zooplankton size distribution, the latter two quantified by the size-abundance power law exponent (see Materials and Methods).

## RESULTS

We find that diatoms, dinoflagellates, and diazotrophs are particularly prone to large abrupt shifts in biomass (Fig. 2, C and E, and fig. S5). These abrupt shifts occur mostly in subtropical latitudes and include both increases and decreases (fig. S6). For instance, diatom and dinoflagellate biomass both spike in the late 21st century in the South Pacific Subtropical Gyre (SPSG), while each exhibits a biomass drop in different parts of the North Pacific in the 2020s and 2050s, respectively. Coccolithophores also exhibit large abrupt shifts in subtropical latitudes, especially around ∼25°N. In contrast, zooplankton, prokaryotes, and picoeukaryotes are far less prone to abrupt shifts despite exhibiting large changes in biomass over >50% (zooplankton) or >30% (prokaryotes and picoeukaryotes) of the ocean (equatorward of 65°) over the 21st century. The differences between functional groups seen in Fig. 2 are likely due to a combination of how opportunistic a given plankton type is and to what extent it has unique resource requirements to coexist with others. Diatoms are the most opportunistic types in the model (i.e., with the highest maximum growth rates) and thus most likely to shift suddenly and substantially in response to a given environmental change [see, e.g., (*26*)]; dinoflagellates have the next-highest maximum growth rates, followed by coccolithophores, and then picoeukaryotes and prokaryotes. At the same time, diatoms are unique in requiring silica, diazotrophs occupy an excess iron/phosphorus supply niche (*27*), and mixotrophic dinoflagellates require both sufficient nutrients and prey to survive. Thus, these phytoplankton types’ biogeography is, in general, more constrained, and their niches can expand or contract as nutrient supply ratios change in a manner that manifests as an abrupt ecological shift. Repeating these analyses on a control simulation without emissions (in which abrupt shifts are therefore due to natural variability) indicates not only that diatoms and dinoflagellates (as well as diazotrophs) are more inherently variable than other types but also that increasing emission scenarios produce substantial increases in the prevalence of abrupt shifts for all plankton types (as well as increases in the amplitude of these shifts, except for picophytoplankton, which have almost no abrupt shifts in either simulation; table S1).

We also find abrupt shifts in whole-ecosystem properties. Abrupt changes in Shannon’s index are mostly decreases (i.e., shifts to lower diversity), concentrated in the subtropics and driven by the decline of coccolithophore populations (Figs. 2D and 3A; note that this group is not more sensitive to temperature or pH change in the simulations but might be in the real ocean). Abrupt changes in richness are fairly distinct from Shannon’s index despite these both being measures of biodiversity; for instance, richness increases in the SPSG coincide with increases in diatom and dinoflagellate biomass, but richness does not drop in the subtropical North Atlantic where diatom and dinoflagellate biomass and Shannon’s index do (Fig. 3, A and B). This is because neither of these functional types is ever dominant in the SPSG, and as richness does not consider the relative abundance of different organisms, it is more sensitive to the presence of rarer organisms, while Shannon’s index also factors in evenness. Primary production also decreases abruptly around ∼25°N but only sporadically and/or comparatively gradually throughout the rest of the ocean (Fig. 3C). Total phytoplankton biomass shifts largely correspond to primary production shifts (Fig. 3D). In contrast, the phytoplankton size distribution shows at least three strong, coherent shifts toward smaller size classes at different time points, in the Caribbean in the 2070s, off the east coast of Australia in the 2080s, and off the west coast of North America in the 2090s (Fig. 3E). A weaker shift in the phytoplankton size distribution also corresponds with a very large shift in the zooplankton size distribution in the Eastern Pacific in the 2060s (Fig. 3, E and F). Thus, abrupt shifts are not isolated to individual phytoplankton types but are substantial enough to cause meaningful shifts in whole-ecosystem properties. Comparison to the control simulation also indicates that the increase in the prevalence of abrupt shifts due to emissions is considerable for each ecosystem property and that these shifts’ amplitude also increases (table S1). However, the response to climate change is complex, with different ecosystem properties shifting at different times and places with different signs and magnitudes. In other words, we find a mosaic of abrupt shifts rather than evidence of a large whole-ocean-ecosystem tipping point, implying greater potential for adaptation of both natural and human systems to changing ecosystem structure over time and space.

Figure 3G summarizes the locations of the abrupt shifts in different ocean locations. The mid-latitudes and the equator are almost entirely devoid of abrupt shifts, and abrupt shifts in the Indian Ocean only occur in a narrow latitudinal band around ∼25°S. In contrast, abrupt shifts occur in multiple ecosystem properties and components through large contiguous regions of the subtropical North and South Atlantic and Pacific. These regions of the ocean thus appear to be prone to substantial, rapid marine microbial ecosystem shifts, particularly in the second half of the 21st century (see also fig. S7). We note that these regions are disjoint from the hots pots of diversity associated with high eddy kinetic energy (*28*), suggesting that a high degree of lateral mixing may mitigate large abrupt shifts. The number of abrupt shifts at a given location (Fig. 3G) is weakly positively associated (*r*^2^ = 0.11, *P* ≪ 0.01) with the difference in community composition between the end and beginning of the century (Fig. 2G), although the regions with the most abrupt shifts have comparatively low Bray-Curtis dissimilarities (≤0.2). This underscores that while community composition changes as a result of abrupt shifts, a great deal of it also happens as a result of gradual changes, particularly at mid-latitudes.

The abrupt shifts can be both positive (increases) and negative (decreases) and have very distinct magnitudes. For diatoms, dinoflagellates, zooplankton, and coccolithophores, roughly equal numbers of positive and negative abrupt shifts occur (fig. S8). Most prokaryotes’ abrupt shifts are positive, and most picoeukaryotes’ abrupt shifts are negative, but these are comparatively rare, as discussed above. The increasing abrupt shifts for diatoms, dinoflagellates, and zooplankton are much larger, in relative terms, than the decreasing ones (fig. S9). For whole-ecosystem properties, both positive and negative abrupt shifts in richness and phytoplankton and zooplankton size distributions are fairly common, and positive abrupt shifts tend to be larger in relative terms than negative ones. However, ≥80% of abrupt shifts in the Shannon index, primary production, and phytoplankton biomass are decreases, and decreases in these latter three quantities are also larger in magnitude than those of richness or phytoplankton and zooplankton size.

To investigate the possible links between ecological abrupt shifts and those in environmental drivers, we analyzed surface temperature and nutrients according to the same procedure. Temperature shifts gradually through almost the entire ocean, with the only abrupt changes occurring in the Labrador Sea (fig. S10), likely driven by changes in deep water formation and not relevant here. Phosphorus, silicate, and nitrate concentrations shift abruptly in much of the ocean but are not clearly coincident with ecosystem properties (fig. S11). Only ≤4% of the abrupt shifts in any ecosystem property are preceded (within 5 years) by an abrupt shift in any nutrient (fig. S12), with the strongest link between silicate and diatoms. However, the shifts in several ecosystem properties (especially drops in primary production and zooplankton biomass) along ∼25°N in the central and eastern North Pacific coincide with a sharp increase in iron concentrations. Nutrient and plankton concentrations interact and partially codetermine each other; the decline in primary production is driven by a poleward shift in the North Pacific Subtropical Gyre’s edge due to changes in macronutrient supply (*29*), which both drive the decrease in zooplankton biomass and allow iron to accumulate, as it is no longer the limiting nutrient. Together, it appears that the abrupt ecological shifts are nonlinear responses to gradual forcing because they are, by and large, not preceded or accompanied by sudden changes in environmental drivers. Thus, it is likely that ecosystem dynamics produce these abrupt shifts, as different phytoplankton types’ competitiveness changes with environmental conditions.

To further explore which components of environmental forcing are generating these nonlinear ecosystem responses, we explored the relationship between the likelihood of observing an abrupt shift and a suite of 13 potential drivers (Materials and Methods, fig. S13, and table S2). We consider (i to iv) supply rates of dissolved organic nitrogen *S_N_*, phosphorus *S*_P_, silicate *S*_Si_, and iron *S*_Fe_, respectively, as one might hypothesize that abrupt shifts occur as a result of crossing a critical nutrient supply threshold; (v to x) the six ratios of these four supply rates (e.g., *S*_N/P_), as one might hypothesize that abrupt shifts occur as a result of crossing a critical nutrient supply ratio threshold; (xi) temperature, as one might hypothesize that abrupt changes are purely associated with higher growth rates and therefore increasingly likely at higher temperatures due to the Eppley curve; (xii) temporal changes in the latitudinal gradient in temperature, as one might hypothesize that abrupt changes are due to the changing positions of ocean fronts; and (xiii) temporal changes in the zooplankton-to-phytoplankton (biomass) ratio, as one might hypothesize that changes in grazing pressure drive abrupt shifts.

Although there are likely many reasons for shifts that occur sporadically, only one of these driver variables shows systematic relationships with biomass or whole ecological properties’ likelihood of abrupt shifts. The likelihood of an abrupt shift occurring increases strongly, for all phytoplankton except prokaryotes and for all whole-ecosystem properties, as *S*_Si_ decreases (and, hence, also as *S*_Si/N_, *S*_Si/P_, and *S*_Si/Fe_ decrease; see fig. S13). This suggests an explanation for many of the abrupt shifts in ecological properties. As silicate supply rate shifts below or above a critical value relative to the supply rate of other nutrients, diatom relative competitiveness is substantially altered (*22*). In these circumstances, a small (although still gradual) change in silicate supply can modulate the extent to which diatoms compete with other phytoplankton for other resources. This switch can reverberate through the plankton community, potentially leading to abrupt shifts in other groups. This underscores that alterations in species that are more susceptible to abrupt shifts, due to, e.g., unique resource requirements or other factors not investigated here such as high sensitivity to ocean acidification, can drive other large changes in the community through their appearance/disappearance. Abrupt shifts in other plankton types or whole-ecosystem properties do not necessarily need to be associated with abrupt shifts in diatoms; either <15% of abrupt shifts in all other ecosystem properties coincide with or are preceded in the previous year by, abrupt shifts in diatoms (except for picoeukaryotic biomass, for which abrupt shifts are comparatively rare). Instead, lower *S*_Si_ rates are strongly associated with higher relative rates of change of diatom biomass ∂*_t_*(diatoms), such that the relative likelihood-*∂_t_*(diatoms) relationship mirrors the relative likelihood-*S*_Si_ relationship (fig. S13 and table S2). This implies that, by and large, abrupt shifts result from small changes in silica supply affecting the extent to which silica-limited diatoms compete with other phytoplankton for other resources. (We also find that the comparatively rare abrupt shifts in prokaryotes and picoeukaryotes are associated with higher-than-average rates of change in the zooplankton-to-phytoplankton ratio, but these are too rare to resolve systematic relationships confidently.)

**Fig. 4.**
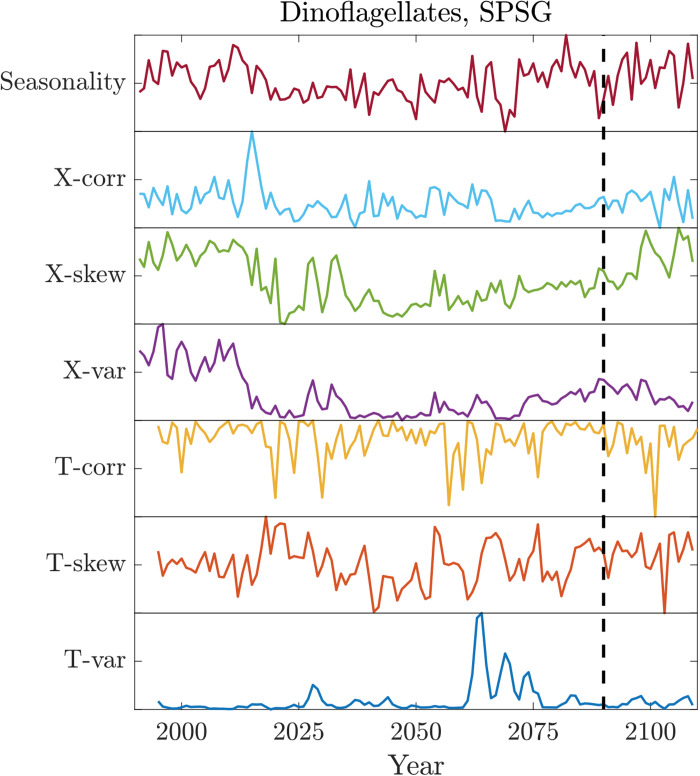
Example of EWS metrics. Time series of different spatial (X-) and temporal (T-) EWS metrics (correlation, variance, and skewness) evaluated from annual output, plus seasonal amplitude evaluated from monthly output, for an example time series with a regional abrupt shift: dinoflagellate biomass in the SPSG. The vertical dashed line indicates timing of abrupt shift.

Although abrupt shifts will occur for different reasons, these results suggest that a key component is the internal nonlinear ecosystem dynamics. These internal dynamics are not necessarily preceded by abrupt changes in environmental variables, suggesting that they cannot be predicted from abrupt shifts in, or EWSs of, environmental variables such as temperature. We find that a suite of standard EWS metrics, applied on an annual time scale, are incapable of predicting these abrupt ecological shifts. EWS metrics are most applicable in cases where gradual environmental forcing pushes a system toward a tipping point, as is the case in this study, but nevertheless, we find that EWSs do not foreshadow abrupt ecosystem shifts. We tested seven standard spatial and temporal EWS metrics (Materials and Methods) on the six major abrupt shifts in the model where multiple ecosystem properties shift simultaneously over large contiguous regions: in the Southeast Pacific (2090s: diatoms, dinoflagellates, and richness), Southwest Pacific [2080s: diatoms, dinoflagellates (illustrated in Fig. 4), phytoplankton size distribution and biomass, primary production, and Shannon’s index], Northeast Pacific (2090s: dinoflagellates, zooplankton, phytoplankton size distribution and biomass, primary production, and Shannon’s index), Northwest Pacific (2060s: diatoms, phytoplankton, and zooplankton size distributions), North Atlantic (2070s: diatoms, dinoflagellates, coccolithophores, primary production, phytoplankton biomass, and Shannon’s index), and South Atlantic (2040s: dinoflagellates, phytoplankton and zooplankton size distributions, and primary production). In all cases, no EWS preceded any of these abrupt shifts. One possible exception is coccolithophore biomass in the North Atlantic, for which two of the seven EWS metrics were triggered a decade before the identified abrupt shift (fig. S14). However, this is likely due to how the abrupt shift timing is calculated; coccolithophore biomass drops and then recovers in the North Atlantic before the multiproperty ecosystem abrupt shift occurs (Fig. 1). On the whole, we do not find EWSs to be effective predictors of marine planktonic ecosystem shifts. This could occur for many reasons, including that crossing a nutrient supply threshold does not produce critical slowing down–type fluctuations, the high dimensionality of the ecosystem, large seasonality of environmental drivers, underlying spatial heterogeneity, spatial processes such as disturbance and dispersal, or coarse temporal resolution of the data (*16*). If any of the first four, this suggests that EWS may not presage abrupt ecological shifts in real-world marine microbial ecosystems, as these factors are even more pronounced in reality than in the model. If the coarseness of model temporal resolution is affecting EWS, then this suggests that submonthly resolution observations may be needed, as even minimal temporal averaging may mask the dynamics of bifurcating systems and render them indistinguishable from stochastic systems (*30*). Other EWS, such as those based on machine learning (*31*), may also prove useful, although they have not yet been developed for, or applied to, the sort of ecological transitions of interest here.

## DISCUSSION

In summary, our results suggest that marine microbial ecosystems are susceptible to large, rapid, unpredictable changes as a result of climate change. While most ecosystem properties of interest such as the biomass of different plankton groups, primary productivity, size distribution, and biodiversity are all expected to change appreciably over much of the ocean during the 21st century, subtropical regions appear substantially more likely to experience rapid shifts rather than gradual changes. This is especially true of the edges of subtropical gyres. This is also true of polar regions (see, e.g., fig. S4), although given the limitations of the model, we do not focus on these. That most of these abrupt shifts occur in the latter half of the 21st century suggests that a less severe emission scenario would lead to substantially fewer abrupt shifts (fig. S7). These rapid shifts appear to be neither preceded by early warning signals nor the result of rapid changes in environmental conditions but, given their spatial patterns, may be most closely linked to the ongoing expansion of subtropical gyres (*29*), particularly in the Northern hemisphere and the Southeast Pacific. Abrupt shifts are increasingly likely with lower silicate supply ratios, which are associated with increased rates of change in silica-limited diatom populations, which can have knock-on effects for the entire plankton community via changes in the extent to which diatoms compete for other resources. Smaller phytoplankton, better adapted to low-nutrient environments and more ubiquitous, have shifts that are almost exclusively gradual in nature. Types that are more opportunistic and/or have more constrained biogeography due to specific resource requirements (e.g., diatoms, dinoflagellates, and diazotrophs) have the most prominent abrupt shifts. The types of systems that are prone to rapid shifts thus appear to be those where niches for these organisms can comparatively easily dis/appear. Given that these shifts occur across a wide range of temperature, nutrient conditions, and ecosystem types and at different times, our results suggest that there is no simple or single (e.g., temperature) threshold that must be crossed for an abrupt shift to occur. Our results likely represent a lower bound on the future of real marine microbial ecosystems, because the latter are far more complex and variable than current computational limitations permit us to simulate, with dynamics that are also more nonlinear. We use empirically informed maximum growth rates, but in the ocean, each functional type will necessarily include some organisms with higher maximum growth rates than the rates that we use, further suggesting that our results may be a conservative indicator of the occurrence of abrupt shifts. On the other hand, our findings are not guaranteed to be conservative indicators of the occurrence of abrupt shifts because, in the ocean, each functional type may also include some organisms with lower maximum growth rates than the rate used in the model, in part because laboratory-based empirically informed maximum growth rates may be overestimates. Ultimately, integrating the evolution of maximum growth rates into plankton ecosystem models will be critical to improving projections of plankton biogeography, including abrupt shifts, with climate change. For natural populations, the maximum growth rate and/or having unique resource requirements may be considered an indicator of propensity toward abrupt shifts, although we strongly caution that further research is needed to explore these links in natural systems. Similarly, diagnosing early warning signals from observations of real marine microbial ecosystems also involves additional pitfalls, most notably measurement error. Abrupt changes in subtropical plankton communities will have knock-on effects for subtropical ocean food webs and for biogeochemical cycling in the subtropics (e.g., the rapid accumulation of iron in the subtropical North Pacific following a decline in primary production there). We thus conclude that it is critical to continue to monitor these marine microbial systems through both synoptic remote sensing and through field programs such as the Hawai’i Ocean Time-series (*32*) and the Bermuda Atlantic Time Series (*33*) to capture possible climate change–driven abrupt shifts. It is also essential to continue to improve our understanding and characterization of marine microbial ecosystems, and early warning signals for them, so that anthropogenically driven changes over these massive ocean regions may be either predicted or avoided.

## MATERIALS AND METHODS

We use the ecosystem model described in (*18*) integrated over a 21st-century climate change scenario (*25*). The ecosystem model resolves the cycling of carbon, phosphorus, nitrogen, silica, iron, and oxygen through inorganic, living, dissolved, and particulate organic phases. It includes 35 phytoplankton across six biogeochemical functional groups (2 prokaryotes, 2 picoprokaryotes, 5 coccolithophores, 5 diazotrophs, 11 diatoms, and 10 mixotrophic dinoflagellates) and 16 zooplankton types. Together, these cover a size range of 0.6 to 2425 μm equivalent spherical diameter (ESD; figs. S1, S10, and S11). Functional groups have distinct allometric relationships for growth, grazing, and sinking parameters following (*22*) and (*34*). Mixotrophs and zooplankton graze on plankton 5 to 15 times smaller than themselves, following a Holling III parameterization (*35*). This ecosystem model has been used and evaluated in current-day simulations in different physical setups (*18*, *36*, *37*). Here, this model ecosystem is forced by output from the Massachusetts Institute of Technology Integrated Global System Model (IGSM) (*24*, *25*, *38*). This Earth system model of intermediate complexity includes coupled atmospheric physics, dynamics and chemistry, and terrestrial and ocean components.

The coupled system is spun up for 2000 years (using 1860 conditions) before simulating 1860 to 2100 changes. Observed concentrations of greenhouse gases, ozone and aerosols, including volcanic stratospheric aerosols, and solar irradiance are used to force the IGSM from 1860 to 2000, and 21st century climate simulations are driven by anthropogenic emissions simulated by the human activity model. In this study, because of the high computational demand of the ecosystem numerical model, we use a single climate simulation from an ensemble of perturbed physics (climate sensitivity), perturbed initial conditions, and varied emission scenarios (*25*). We focus on the climate simulation with a medium climate sensitivity (3.0°C) under a high emission scenario similar to the Representative Concentration Pathway 8.5 used in the Coupled Model Intercomparison Project 5. The ocean component is the MITgcm (*39*) with a 2°-by-2.5° resolution grid and 22 vertical layers (10-m thickness at the surface to 500 m at the bottom). The ocean physics displays a realistic year-to-year variability in surface temperature and produces interannual variability (e.g., El Niño–Southern Oscillation) with frequency, seasonality, magnitude, and patterns in general agreement with the observations (*25*).

Here, we use the output from this ocean component to drive the ecosystem model. Because of the computational expense, we spin up the ecosystem for 100 years, using preindustrial control fields, before starting the transient climate simulation (1860–2100). A repeating seasonal cycle was quickly reached, as well as stable thermocline nutrients. There was only a small biogeochemical drift associated with upwelling of deep water. The several thousand years of integration needed to adjust the deep ocean was computationally unfeasible with the full ecosystem model. We do however conduct a control simulation from the same initial conditions but with no emissions for 240 years to establish that any remaining drifts are small and do not affect the results presented here. The abrupt shifts in the control simulation are encouraging, as they indicate that the model is complex enough to simulate a substantial amount of ecological variability as expected in the real ocean and because the difference in the prevalence of abrupt shifts between different variables in the control simulation is sensible. The same IGSM ocean component and ecosystem coupling/spin-up procedure was used in a previous study with an ecosystem of lower complexity (*24*). The ecosystem model, within different physical frameworks, has been evaluated in several recent papers (*18*, *36*, *37*, *40*). Similar to these studies, the IGSM-driven ecosystem for current day (2000–2020) captures the patterns of high and low chlorophyll a (Chl-a) values between upwelling high-latitude, equatorial, and nutrient-limited subtropical zones seen in satellite-derived Chl-a (fig. S12, top row). The model does underestimate Chl-a (and probably biomass) in some patches in the subtropical gyres and overestimates in the mid-high latitudes (Fig. 2B). However, we note that satellite-estimated Chl-a have large uncertainties (*41*), especially in the Southern Ocean (*42*). The low Chl-a patches in the subtropics is likely due to the lack of explicit mesoscale features [due to the low model resolution; see, e.g., (*43*)] that would help in the supply of nutrients in these oligotrophic regions. The model does not adequately capture sea-ice and sea-ice edge algae and thus does not represent the polar regions well. Similar model versions have been extensively compared to functional groups (*18*, *21*, *22*), and the model formulation used here captures the observed ubiquitous picophytoplankton and the more constrained patterns of nano- and microphytoplankton (fig. S3), making it well suited to investigate the dynamics explored here. The model tends to overestimate the Chl-a in nanophytoplankton at the expense of the microphytoplankton. However, we note the relatively arbitrary cutoff between the two size classes; if, in our model setup (fig. S1), we shifted to a cutoff of 3 μm, then we would compare substantially better. We also compare the model functional group distribution to a compilation of observations [MAREDAT database; (*1*) and references therein; fig. S3]. Although the observations are sparse, we capture that the universal nature of the picophytoplankton also noted, in this dataset, the more bounded domain of the diazotrophs (including observed lack of diazotrophs in the South Pacific gyre), and the pattern of enhanced diatom biomass in high latitudes and low in subtropical gyres. The diazotroph population in the Southern Ocean is unrealistic. We also appear to overestimate the coccolithophore biomass relative to MAREDAT in many regions; however, the conversion from cells to biomass in that compilation was estimated to have uncertainties as much as several 100% (*44*). The MAREDAT compilation did not include a category for dinoflagellates. Because of sea ice retreat and polar amplification, latitudes >65° are rife with large abrupt changes mostly corresponding to range expansions (fig. S4). Although abrupt changes in polar regions are indeed very likely to occur and be important, we do not focus on them, as the model does not adequately capture sea-ice algae and other relevant ice-edge phenomena. Here, instead, we focus on locations equatorward of 65°.

All analyses are performed on depth-integrated biomass (over the full water column), although surface plankton are similarly prone to abrupt shifts. Biomass of each functional group is the sum of the biomasses of all plankton within that group (i.e., summed over size). Bray-Curtis dissimilarity BC for each grid cell from 1990–2000 and 2100–2110 is calculated by

$$\begin{array}{c} \text{BC}=1-2\sum_{i}\text{min}\;(B_{1990-2000}^{i},B_{2100-2110}^{i})/\\ \sum_{i}(B_{1990-2000}^{i}+B_{2100-2110}^{i}) \end{array}$$

where $B_{t}^{i}$ is the average biomass for plankton type *i* over year(s) *t*. Shannon index *H* is calculated by the standard formula of *H_t_* = − ∑*_i_B_i_* ln *B_i_*. Richness is calculated as the number of plankton types contributing more than 0.1% to total biomass. The size distribution α is calculated by estimating the slope of ln(biomass) versus ln(ESD) for size classes contributing more than 0.1% to total biomass using the robust Theil-Sen method and adding three; this is equivalent to regressing abundance versus ESD, as model plankton densities are size independent. Size exponents were quantitatively similar, and exhibited similar spatial patterns, to ordinary least squares regression, but the Theil-Sen estimator is preferred because it is insensitive to outliers that often occur when taking logarithms of small numbers.

Abrupt shifts were identified by comparing, for an annual time series *x*(*t*) from 1990 to 2110 of each ecosystem property at each grid point, a step-function model versus a linear model. The three-parameter step-function model [i.e., *x*(*t*) = α + β*H*(*t* − τ) + ε, where (α, β, τ) are model parameters and ε is the error] was compared with the two-parameter linear model [i.e., *x*(*t*) = *a* + *bt* + ε] using the Akaike information criterion (AIC) (*45*) and assuming Gaussian errors, i.e., the model with the lower AIC = 2*k* + *N* ln (RSS/*N*) was selected, where *k* is the number of model parameters, *N* is the length of the time series, and RSS is the residual sum of squares. As we are interested in the largest shifts, we require ∣β∣/α > 0.25, i.e., a shift of larger than 25%, for phytoplankton functional type biomasses and surface nutrient concentrations (Fig. 2 and fig. S6), and ∣β∣/α > 0.1, i.e., a shift of larger than 10%, for whole-ecosystem properties and temperature (Fig. 3 and fig. S5). We used a larger threshold for individual phytoplankton types than for whole-ecosystem properties because the total ecosystem is necessarily less variable than its component parts. Our results are quantitatively but not qualitatively dependent on these ad hoc threshold choices (fig. S15). We identify change points using MATLAB’s findchangepoints function, which implements the algorithm described in (*46*). This finds the best-fitting τ above. We then evaluate the amplitude of the change between these constant values (β − α above) relative to the initial value α and evaluate the goodness of fit of this three-parameter step function (i.e., *t*, *c*_0_, and *c*_1_) relative to a (two-parameter) linear trend using the AIC (*45*). We discard all change points that correspond to differences below the chosen amplitude threshold or where a linear trend explains the time series better, where the AIC also accounts for the fact that the linear trend has fewer free parameters.

For regions with large, synchronous abrupt shifts in multiple ecosystem properties, we evaluated both spatial and temporal EWS metrics’ ability to presage these shifts. We identified contiguous regions in the subtropical South and North Pacific and Atlantic where four or more ecosystem properties shifted within a 5-year period. We then computed, for each property and location, a time series of seven standard EWSs: (i) temporal variance, i.e., the variance of that property over a 5-year moving window, (ii) temporal skewness, (iii) temporal correlation, i.e., the lag-1 correlation of the time series over the same moving window, (iv) spatial correlation, i.e., the correlation of time series with their neighboring grid points at each time step, (v) spatial variance, i.e., the variance over the region at each time step, (vi) spatial skewness, and (vii) the seasonal amplitude, i.e., the difference in the monthly maximum and the monthly minimum of the time series within each year. The first six of these are not an exhaustive list of EWS metrics but include all of the general phenomena of rising memory (iii), rising variability/flickering (ii), spatial slowing down–based indication (iv), and spatial variability–based indication (vi) (*12*). The seasonal amplitude (vii) was included to assess whether subannual variability might be a useful EWS. EWS results were not sensitive to either the bounds of the selected spatial region or the temporal window size, and the next higher order moment (i.e., kurtosis) behaved similarly both for temporal and spatial EWS.

Because the rates of change of the environmental drivers are slow relative to the plankton dynamics in the model, the abrupt shifts described here are unlikely to be due to rate-dependent effects. Therefore, reanalyzing the model over different time windows from the initial year up to a variable end year should produce qualitatively similar abrupt shifts as lower emission scenarios run to 2100. We do so to use our simulation to evaluate the possible impact of lower emission scenarios. Figure S7 shows, for each ecosystem property, the fraction of abrupt shifts detected when analyzing years 1990–20XX for each year from 2000 to 2099, relative to 2100.

To maximize the extent to which abrupt shifts are related to multidecadal/centennial changes (or, equivalently, to minimize the extent to which they are due to interannual variability), we do not allow for multiple change points in analyzing each time series. To test for the potential impact of this choice on our conclusions, we test for the possibility of sequential complementary abrupt shifts. We repeat our analyses but fit a model where multiple abrupt shifts of the same sign are allowed (i.e., two large abrupt shifts that cancel each other out are neglected). We find that 19% of all the abrupt shifts in all ecosystem properties considered here are accompanied by additional large, abrupt shifts at the same locations but in different years. We consider this frequency justifiably low to exclude consideration of these secondary shifts, as they would not affect our conclusions, especially considering that 40% of all secondary abrupt shifts are in diazotroph biomass, which we do not focus on in the main text. Analysis of the control simulation shows that the natural variability can still produce multidecadal/centennial-scale abrupt shifts but that these are smaller and much rarer than in the emission scenario simulation (table S1).

Another common information criterion is the Bayesian (or Schwarz) information criterion (BIC), which is more conservative in that it favors models with fewer parameters than the AIC (*47*). To test the sensitivity of our results to our choice of fitting metric, we repeated our analyses with the BIC. Less than 10% of all abrupt shifts were excluded by this stricter metric, indicating that our conclusions are insensitive to this choice; the most sensitive ecosystem property was primary production, where the fraction of ocean area equatorward of 65° with abrupt shifts decreased by 16% when using the BIC.

For fig. S13 and table S2, the relative likelihood of an abrupt shift occurring is computed as the ratio of the probability density function of the driver variable at places and times where abrupt shifts occur to the probability density function of that driver variable for the ocean overall, so a relative likelihood of *L* for a given ecological property (such as biomass or species richness) and a given value of a given driver variable (such as a supply ratio of nitrogen to phosphorous of 16) means that abrupt shifts in that property are *L* times as likely as average to occur when and where that driver variable is equal to that value. *∂_t_* refers to the difference between a given year and the previous year, and *∂_y_* refers to the centered difference approximation of the latitudinal gradient. Relative changes in diatoms and the zooplankton-to-phytoplankton ratio are normalized by the values in the previous year. As with nutrient concentrations and temperature in figs. S10 and S11, the temperature at and nutrient supply to the topmost grid cell are used. Nutrient supply rates are calculated as the summation of remineralization of organic matter, transport and mixing of the nutrient, and (in the case of iron) dust deposition.

## Supplementary Materials

### This PDF file includes:

Figs. S1 to S15 Tables S1 and S2 References
