# Supplementary material for: Abrupt shifts in 21st-century plankton communities
Source: Sci Adv. 2021 Oct 29;7(44):eabf8593. doi: 10.1126/sciadv.abf8593 (PMC8555899; doi:10.1126/sciadv.abf8593)
Supplement: Supplementary file 1 — Figs. S1 to S15 Tables S1 and S2 References [file sciadv.abf8593_sm.pdf]

Science Advances
AAAS

# Supplementary Materials for

## Abrupt shifts in 21st-century plankton communities

B. B. Cael*, Stephanie Dutkiewicz, Stephanie Henson

*Corresponding author. Email: cael@noc.ac.uk

Published 29 October 2021, *Sci. Adv.* **7**, eabf8593 (2021)
DOI: 10.1126/sciadv.abf8593

**This PDF file includes:**

Figs. S1 to S15
Tables S1 and S2
References

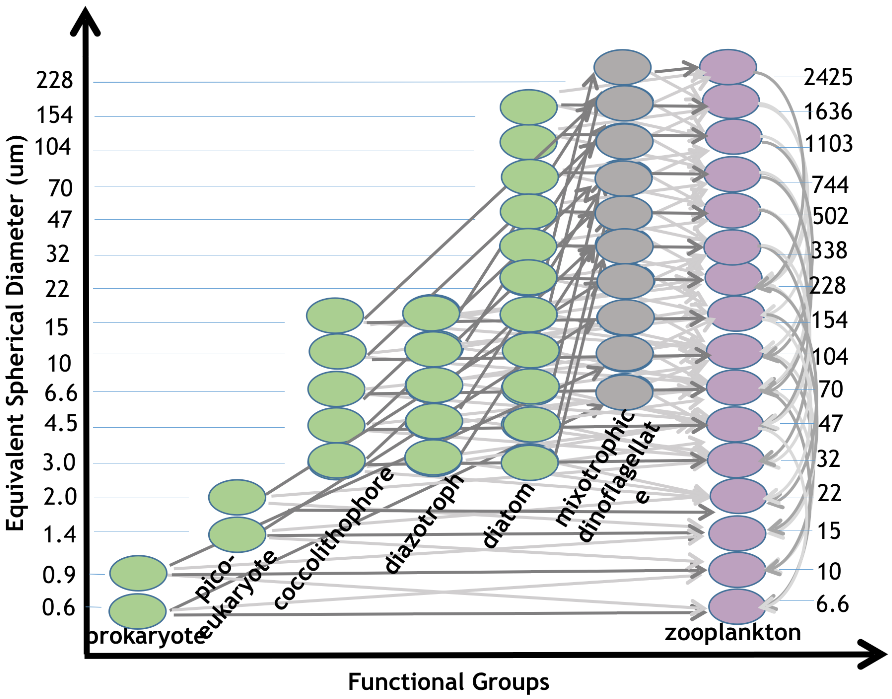


Figure S1: Schematic of model plankton by size and function.

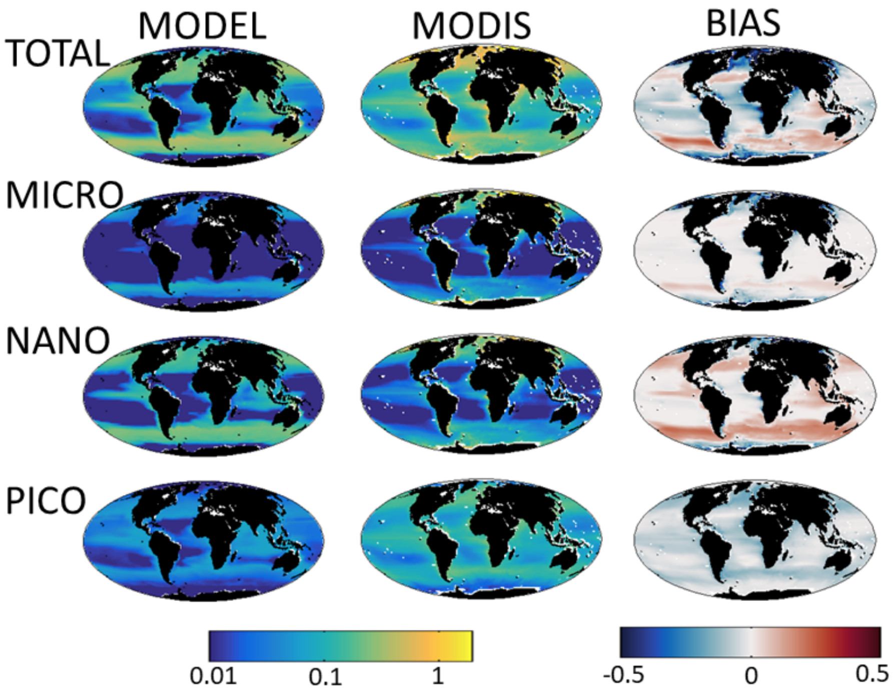


Figure S2: Comparison of Annual Mean Surface Chl-a (mg Chl / $m^3$). (Top row) total Chl-a; and Chl-a in size classes (Second row) micro (>20$\mu$m); (Third row) nano (2-20$\mu$m); (Bottom row) pico (<2$\mu$m). (Left column) Model 2000-2020 mean; (Middle Column) Satellite Observations, top from NASA MODIS, other three panels are the satellite-based estimates from Ref. (*19*); (Right Column) Model bias determined as model minus observations. The middle column shows mean of all available satellite measurements, with missing observations in the polar winters and under clouds; while model results are mean (0-50m).

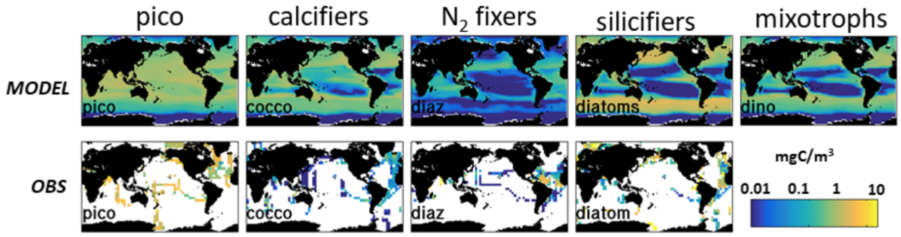


Figure S3: Comparison to Observations of Functional Groups. (top) mean from 2000-2020 from model (0-60m) and (bottom) data compilation (MAREDAT) (*20*) in carbon biomass (mg C / $m^3$). For the MAREDAT databases: pico-phytoplankton (*48*); coccolithophores (*44*); diazotrophs (*49*); diatoms (*50*). There was no MAREDAT dataset for dinoflagellates. We do not show biases as these would not be reasonable comparisons since MAREDAT data presented here combines all measurements regardless of time, while model show annual mean.

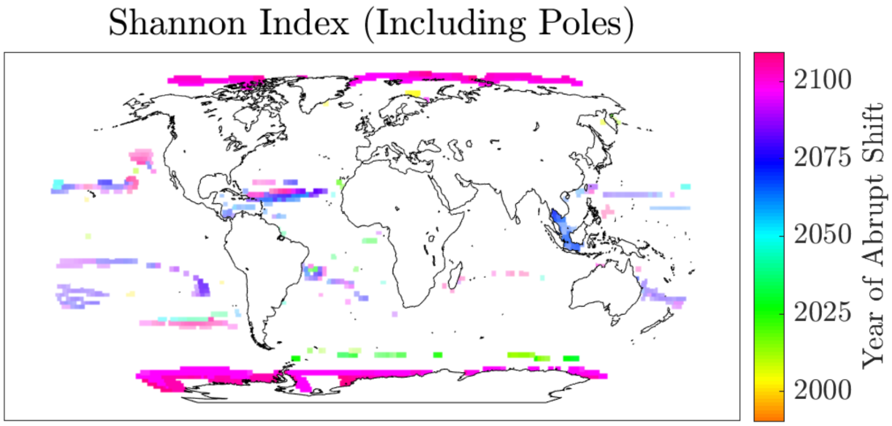


Figure S4: Same as Figure 3a but including poles.

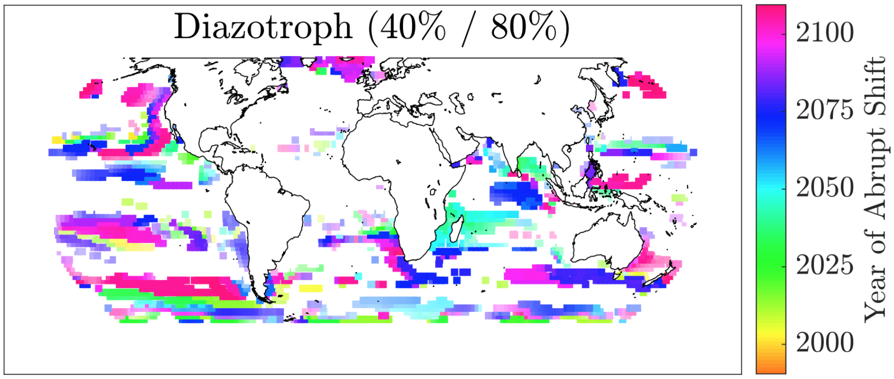


Figure S5: Same as Figures 2a-f for diazotrophs.

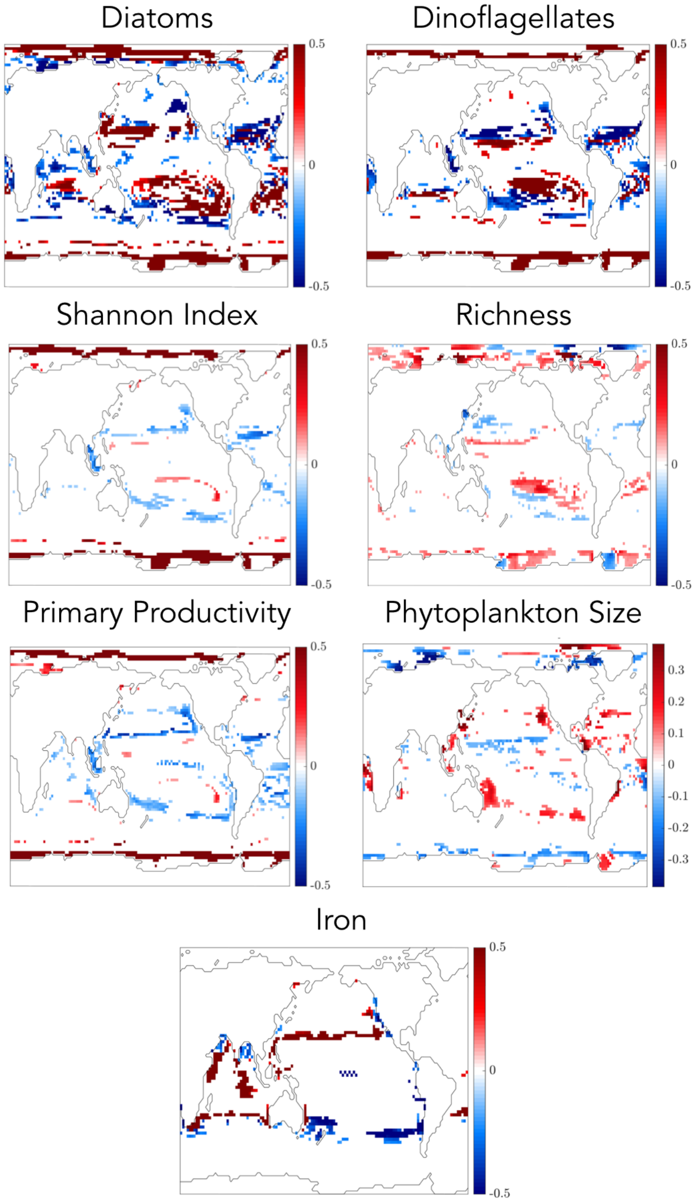


Figure S6: Same as maps in Figures 2a-f, 3a-f, S5, S6 for those properties where the sign of an abrupt change is mentioned in the text. Blue corresponds to declines and red corresponds to increases, with color intensity corresponding to the magnitude of the shift up to saturation at 50%. Red in the phytoplankton size distribution plot corresponds to a shift to smaller size classes (larger magnitude of power-law size distribution exponent).

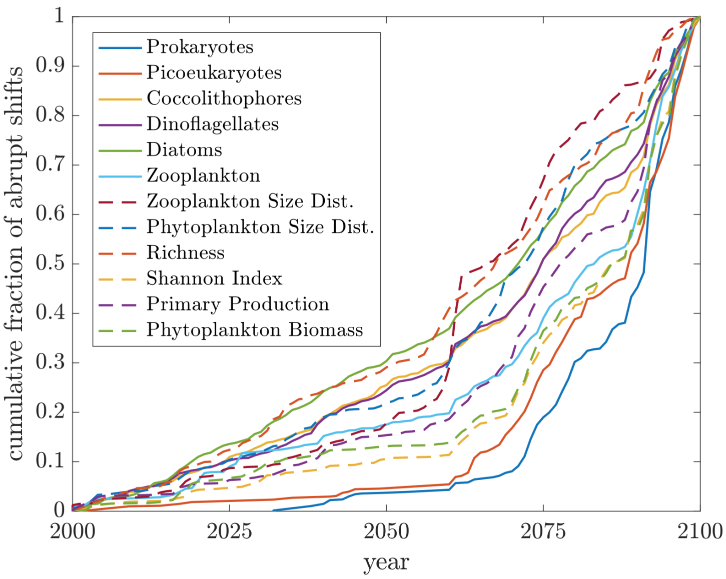


Figure S7: For each ecosystem property, the fraction of 21st-century abrupt shifts identified when analyzing model output from 1990-$y$ as a function of end year $y$ between 2000 and 2100. Depending on the ecosystem property, 70-96% of the abrupt shifts are only identified when analyzing model output including years past 2050.

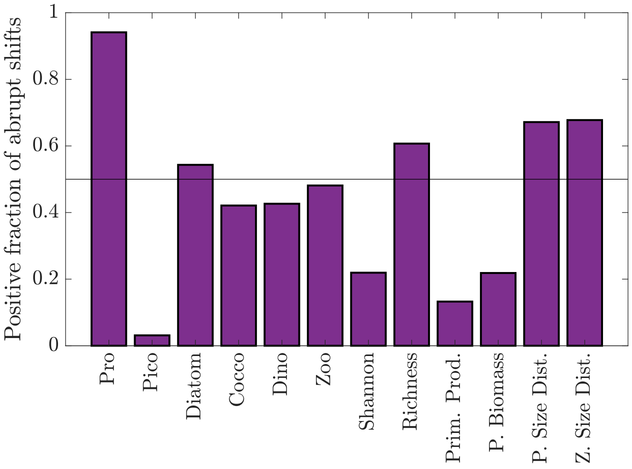


Figure S8: For each ecosystem property, the fraction of 21st-century abrupt shifts identified that are positive, i.e. increases.

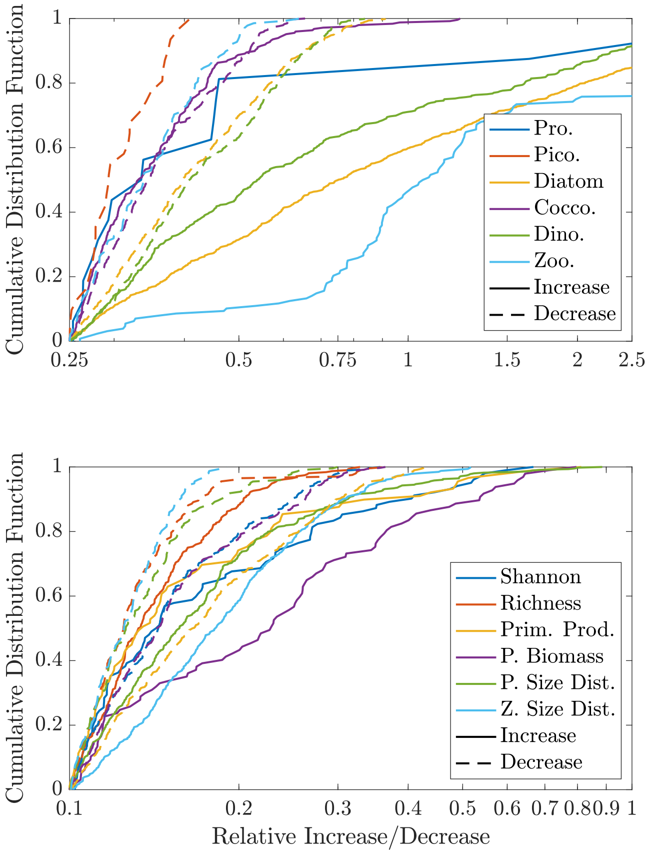


Figure S9: For each ecosystem property, the cumulative distribution of the amplitude of both positive (increasing) and negative (decreasing) abrupt shifts. Negative prokaryotic and positive picoeukaryotic abrupt shifts are not plotted because each is comprised of only a single anomalous grid cell.

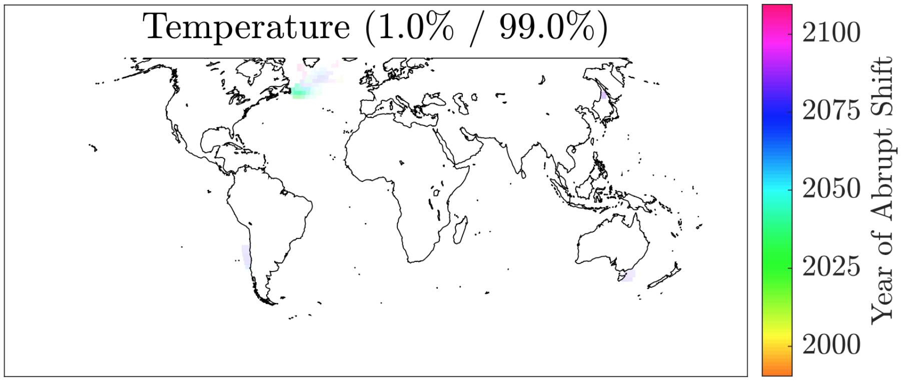


Figure S10: Same as Figures 3a-f for top ocean grid layer temperature.

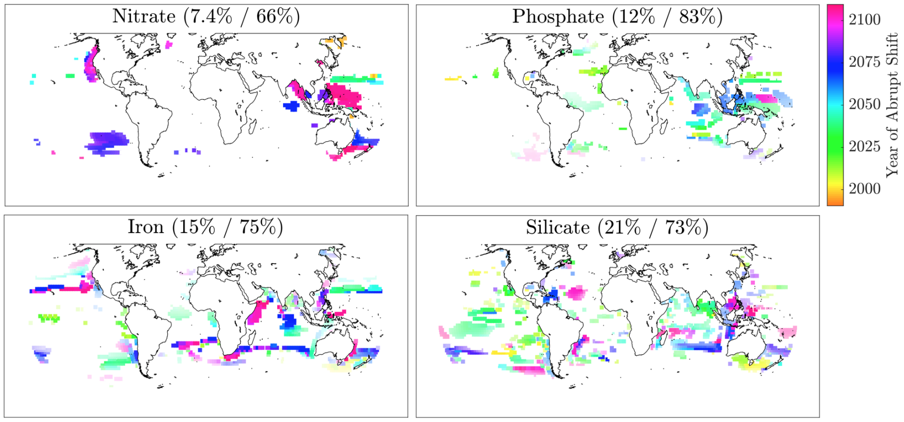


Figure S11: Same as Figures 2a-f for top ocean grid layer nutrient concentrations.

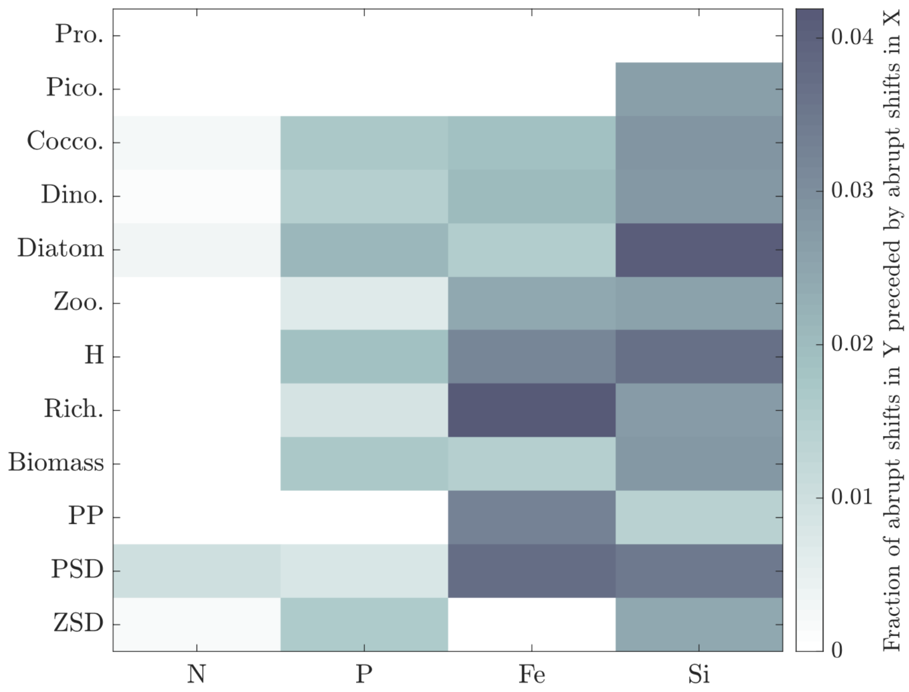


Figure S12: Fraction of abrupt shifts in each ecosystem property preceded (within five years) by an abrupt shift in each nutrient. For instance, in the maximum case, for 4.2% of the locations where an abrupt shift in diatom biomass is detected, an abrupt shift in silicate concentrations is detected 1-5 years beforehand. When using a window of 2 years, these fractions range from 0-0.027; when using a window of 10 years, they range from 0-0.057.

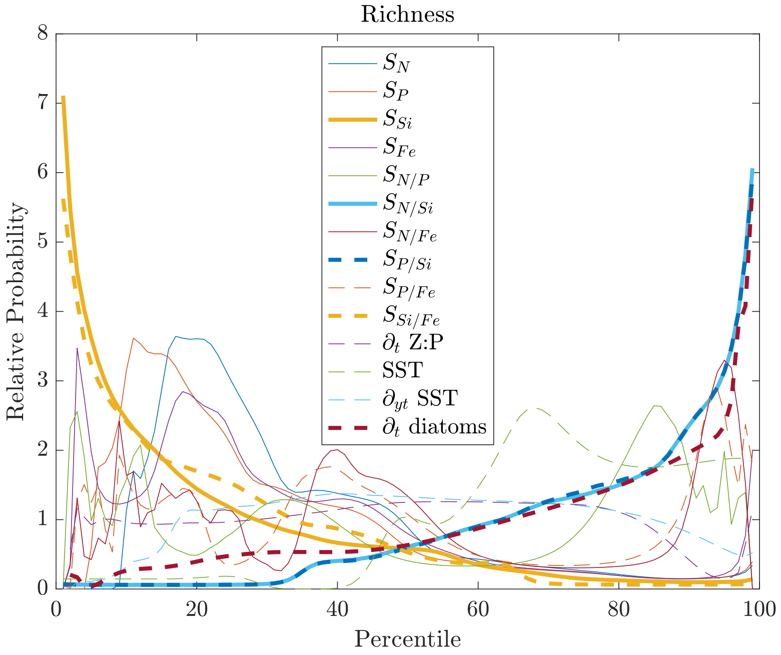


Figure S13: Relative likelihood of an abrupt shift occurring in species richness as a function of different driver variables, and as a function of the relative rate of change of diatom biomass. X-axis refers to the percentiles of the driver variables, so that all drivers can be plotted on a single axis. The only systematic relationship is with silicate supply $S_{Si(/N/P/Fe)}$ and $\partial_t$ diatoms.

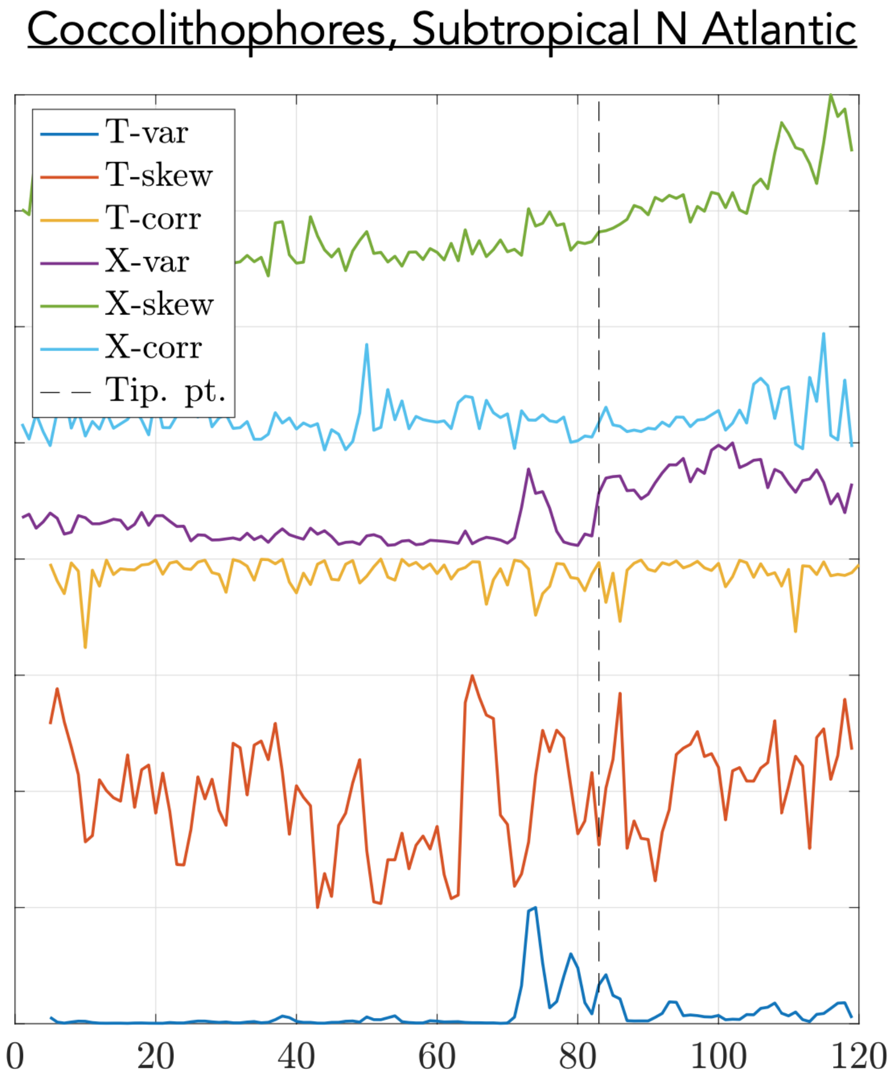


Figure S14: Same as Figure 4 for coccolithophores in the Subtropical North Atlantic. Note the purple and blue variance time series' maxima.

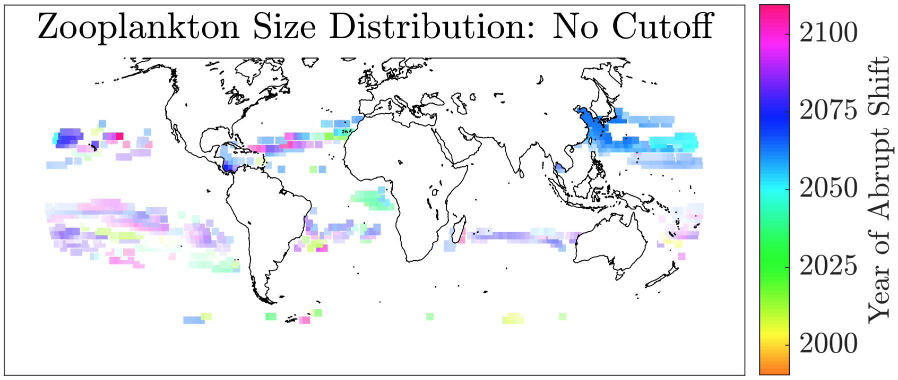


Figure S15: a) Same as Figure 3f. b) Same as Figure 3f without 10% cutoff threshold. Regions with abrupt shifts are slightly expanded but still confined to the subtropics.

Table S1: For each ecosystem property considered here, the fraction of ocean area equatorward of 65° for which abrupt changes are identified (Ext.) is given for both the emissions (E.) scenario simulation and the control (C.) simulation. The third and fourth column give the median amplitude (Amp.) of the abrupt shifts in each case.

| Property | E. Ext. (%) | C. Ext. (%) | E. Amp. (%) | C. Amp. (%) |
|---|---|---|---|---|
| Prokaryotes | 0.30 | 0.015 | 42 | 34 |
| Picoeukaryotes | 1.2 | 0.21 | 82 | 29 |
| Coccolithophores | 12 | 0.69 | 27 | 34 |
| Diazotrophs | 40 | 10 | 37 | 81 |
| Diatoms | 25 | 7.5 | 40 | 51 |
| Dinoflagellates | 20 | 4.1 | 31 | 45 |
| Zooplankton | 5.3 | 1.9 | 32 | 47 |
| Shannon Index | 8.4 | 1.6 | 12 | 14 |
| Richness | 9.2 | 0.93 | 12 | 13 |
| Primary Production | 12 | 4.3 | 14 | 16 |
| Phyto. Biomass | 7.9 | 1.6 | 12 | 15 |
| Phyto. Size Dist. | 12 | 1.8 | 11 | 14 |
| Zoo. Size Dist. | 8.8 | 0.78 | 14 | 15 |

Table S2: For each ecosystem property considered here, whether or not there is a systematic increase in the relative probability of an abrupt change occurring at a given place and time as the supply rate $S_{Si}$ decreases (and as the supply ratios $S_{Si/N}$, $S_{Si/P}$, $S_{Si/Fe}$ decrease, and as the relative change in diatom biomass $\partial_t$(diatoms) increases), or whether that ecosystem property has a systematic relationship with any other putative driver. See also Figure S13. Prokaryotes and Picoeukaryotes are not considered because their abrupt shifts are too rare to resolve systematic relationships confidently, though $\partial_t Z/P$ is higher than average when and where abrupt shifts in these plankton types occur.

| Property | $S_{Si(/N/P/Fe)}$ and $\partial_t$(diatoms) | another putative driver |
|---|---|---|
| Coccolithophores | Yes | No |
| Diatoms | Yes | No |
| Dinoflagellates | Yes | No |
| Zooplankton | No | No |
| Shannon Index | Yes | No |
| Richness | Yes | No |
| Primary Production | Yes | No |
| Phyto. Biomass | Yes | No |
| Phyto. Size Dist. | Yes | No |
| Zoo. Size Dist. | Yes | No |

## REFERENCES AND NOTES


1. E. T. Buitenhuis, T. Hashioka, C. L. Quéré, Combined constraints on global ocean primary production using observations and models. *Global Biogeochem. Cycles* **27**, 847–858 (2013).

2. P. G. Falkowski, T. Fenchel, E. F. Delong, The microbial engines that drive Earth's biogeochemical cycles. *Science* **320**, 1034–1039 (2008).

3. R. G. Williams, M. J. Follows, *Ocean Dynamics and the Carbon Cycle: Principles and Mechanisms* (Cambridge Univ. Press, 2011).

4. S. C. Doney, M. Ruckelshaus, J. E. Duffy, J. P. Barry, F. Chan, C. A. English, H. M. Galindo, J. M. Grebmeier, A. B. Hollowed, N. Knowlton, J. Polovina, N. N. Rabalais, W. J. Sydeman, L. D. Talley, Climate change impacts on marine ecosystems. *Ann. Rev. Mar. Sci.* **4**, 11–37 (2012).

5. J. K. Moore, W. Fu, F. Primeau, G. L. Britten, K. Lindsay, M. Long, S. C. Doney, N. Mahowald, F. Hoffman, J. T. Randerson, Sustained climate warming drives declining marine biological productivity. *Science* **359**, 1139–1143 (2018).

6. S. A. Levin, Ecosystems and the biosphere as complex adaptive systems. *Ecosystems* **1**, 431–436 (1998).

7. K. Faust, J. Raes, Microbial interactions: From networks to models. *Nat. Rev. Microbiol.* **10**, 538–550 (2012).

8. J. M. Drake, B. D. Griffen, Early warning signals of extinction in deteriorating environments. *Nature* **467**, 456–459 (2010).

9. L. Bopp, L. Resplandy, J. C. Orr, S. C. Doney, J. P. Dunne, M. Gehlen, P. Halloran, C. Heinze, T. Ilyina, R. Seferian, J. Tjiputra, M. Vichi, Multiple stressors of ocean ecosystems in the 21st century: Projections with CMIP5 models. *Biogeosciences* **10**, 6225–6245 (2013).

10. B. I. McNeil, R. J. Matear, Southern ocean acidification: A tipping point at 450-ppm atmospheric $CO_2$. *Proc. Natl. Acad. Sci. U.S.A.* **105**, 18860–18864 (2008).

11. S. R. Hare, N. J. Mantua, Empirical evidence for north pacific regime shifts in 1977 and 1989. *Prog. Oceanogr.* **47**, 103–145 (2000).

12. M. Scheffer, J. Bascompte, W. A. Brock, V. Brovkin, S. R. Carpenter, V. Dakos, H. Held, E. H. Van Nes, M. Rietkerk, G. Sugihara, Early-warning signals for critical transitions. *Nature* **461**, 53–59 (2009).

13. M. A. Litzow, J. D. Urban, B. J. Laurel, Increased spatial variance accompanies reorganization of two continental shelf ecosystems. *Ecol. Appl.* **18**, 1331–1337 (2008).

14. A. S. Gsell, U. Scharfenberger, D. Özkundakci, A. Walters, L.-A. Hansson, A. B. Janssen, P. Nõges, P. C. Reid, D. E. Schindler, E. Van Donk, V. Dakos, R. Adrian, Evaluating early-warning indicators of critical transitions in natural aquatic ecosystems. *Proc. Natl. Acad. Sci. U.S.A.* **113**, E8089–E8095 (2016).

15. S. R. Carpenter, J. J. Cole, M. L. Pace, R. Batt,W. Brock, T. Cline, J. Coloso, J. R. Hodgson, J. F. Kitchell, D. A. Seekell, L. Smith, B. Weidel, Early warnings of regime shifts: A whole-ecosystem experiment. *Science* **332**, 1079–1082 (2011).

16. V. Dakos, S. R. Carpenter, E. H. van Nes, M. Scheffer, Resilience indicators: Prospects and limitations for early warnings of regime shifts. *Philos. Trans. R. Soc. B* **370**, 20130263 (2015).

17. T. J. Wagner, I. Eisenman, False alarms: How early warning signals falsely predict abrupt sea ice loss. *Geophys. Res. Lett.* **42**, 10,333–10,341 (2015).

18. S. Dutkiewicz, P. W. Boyd, U. Riebesell, Exploring biogeochemical and ecological redundancy in phytoplankton communities in the global ocean. *Glob. Chang. Biol.* **27**, 1196–1213 (2021).

19. B. A. Ward, Temperature-correlated changes in phytoplankton community structure are restricted to polar waters. *PLOS ONE* **10**, e0135581 (2015).

20. E. T. Buitenhuis, M. Vogt, R. Moriarty, N. Bednaršek, S. C. Doney, K. Leblanc, C. L. Quéré, Y.-W. Luo, C. O'Brien, T. O'Brien, J. Peloquin, R. Schiebel, C. Swan, MAREDAT: Towards a world atlas of MARine Ecosystem DAta. *Earth Sys. Sci. Data* **5**, 227–239 (2013).

21. S. Dutkiewicz, A. E. Hickman, O. Jahn,W. W. Gregg, C. B. Mouw, M. J. Follows, Capturing optically important constituents and properties in a marine biogeochemical and ecosystem model. *Biogeosciences* **12**, 4447–4481 (2015).

22. S. Dutkiewicz, P. Cermeno, O. Jahn, M. J. Follows, A. Hickman, D. Taniguchi, B. A. Ward, Dimensions of marine phytoplankton diversity. *Biogeosciences* **17**, 609–634 (2020).

23. K. Riahi, S. Rao, V. Krey, C. Cho, V. Chirkov, G. Fischer, G. Kindermann, N. Nakicenovic, P. Rafaj, Rcp 8.5—A scenario of comparatively high greenhouse gas emissions. *Clim. Change* **109**, 33–57 (2011).

24. S. Dutkiewicz, A. E. Hickman, O. Jahn, S. Henson, C. Beaulieu, E. Monier, Ocean colour signature of climate change. *Nat. Commun.* **10**, 578 (2019).

25. E. Monier, J. R. Scott, A. P. Sokolov, C. E. Forest, C. A. Schlosser, An integrated assessment modeling framework for uncertainty studies in global and regional climate change: The MIT IGSM-CAM (version 1.0). *Geosci. Model Dev.* **6**, 2063–2085 (2013).

26. R. H. MacArthur, E. O. Wilson, *The Theory of Island Biogeography* (Princeton Univ. Press, 2001), vol. 1.

27. S. Dutkiewicz, B. A. Ward, J. R. Scott, M. J. Follows, Understanding predicted shifts in diazotroph biogeography using resource competition theory. *Biogeosciences* **11**, 5445–5461 (2014).

28. A. D. Barton, S. Dutkiewicz, G. Flierl, J. Bragg, M. J. Follows, Patterns of diversity in marine phytoplankton. *Science* **327**, 1509–1511 (2010).

29. H. Yang, G. Lohmann, U. Krebs-Kanzow, M. Ionita, X. Shi, D. Sidorenko, X. Gong, X. Chen, E. J. Gowan, Poleward shift of the major ocean gyres detected in a warming climate. *Geophys. Res. Lett.* **47**, e2019GL085868 (2020).

30. D. Toker, F. T. Sommer, M. D'Esposito, A simple method for detecting chaos in nature. *Commun. Biol.* **3**, 11 (2020).

31. S.-H. Moon, Y.-H. Kim, Y. H. Lee, B.-R. Moon, Application of machine learning to an early warning system for very short-term heavy rainfall. *J. Hydrol.* **568**, 1042–1054 (2019).

32. D. M. Karl, R. Lukas, The hawaii ocean time-series (hot) program: Background, rationale and field implementation. *Deep-Sea Res. II Top. Stud. Oceanogr.* **43**, 129–156 (1996).

33. A. F. Michaels, A. H. Knap, Overview of the U.S. JGOFS Bermuda Atlantic time-series study and the Hydrostation S program. *Deep-Sea Res. II Top. Stud. Oceanogr.* **43**, 157–198 (1996).

34. B. A. Ward, S. Dutkiewicz, O. Jahn, M. J. Follows, A size-structured food-web model for the global ocean. *Limnol. Oceanogr.* **57**, 1877–1891 (2012).

35. C. S. Holling, Some characteristics of simple types of predation and parasitism. *Can. Entomol.* **91**, 385–398 (1959).

36. A. M. Kuhn, S. Dutkiewicz, O. Jahn, S. Clayton, T. A. Rynearson, M. R. Mazloff, A. D. Barton, Temporal and spatial scales of correlation in marine phytoplankton communities. *J. Geophys. Res. Oceans* **124**, 9417–9438 (2019).

37. M. Sonnewald, S. Dutkiewicz, C. Hill, G. Forget, Elucidating ecological complexity: Unsupervised learning determines global marine eco-provinces. *Sci. Adv.* **6**, eaay4740 (2020).

38. A. P. Sokolov, C. A. Schlosser, S. Dutkiewicz, S. Paltsev, D. W. Kicklighter, H. D. Jacoby, R. G. Prinn, C. E. Forest, J. M. Reilly, C. Wang, B. Felzer, M. C. Sarofim, J. Scott, P. H. Stone, J. M. Melillo, J. Cohen, "The MIT Integrated Global System Model (IGSM) version 2: Model description and baseline evaluation" (Technical Report, MIT Joint Program on the Science and Policy of Global Change, 2005).

39. J. Marshall, A. Adcroft, C. Hill, L. Perelman, C. Heisey, A finite-volume, incompressible Navier stokes model for studies of the ocean on parallel computers. *J. Geophys. Res. Oceans* **102**, 5753–5766 (1997).

40. P. Tréguer, C. Bowler, B. Moriceau, S. Dutkiewicz, M. Gehlen, O. Aumont, L. Bittner, R. Dugdale, Z. Finkel, D. Iudicone, O. Jahn, L. Guidi, M. Lasbleiz, K. Leblanc, M. Levy, P. Pondaven, Influence of diatom diversity on the ocean biological carbon pump. *Nat. Geosci.* **11**, 27–37 (2018).

41. T. S. Moore, J. W. Campbell, M. D. Dowell, A class-based approach to characterizing and mapping the uncertainty of the MODIS ocean chlorophyll product. *Remote Sens. Environ.* **113**, 2424–2430 (2009).

42. R. Johnson, P. G. Strutton, S. W. Wright, A. McMinn, K. M. Meiners, Three improved satellite chlorophyll algorithms for the Southern Ocean. *J. Geophys. Res. Oceans* **118**, 3694–3703 (2013).

43. F. Ribalet, J. Swalwell, S. Clayton, V. Jiménez, S. Sudek, Y. Lin, Z. I. Johnson, A. Z. Worden, E. V. Armbrust, Light-driven synchrony of *Prochlorococcus* growth and mortality in the subtropical pacific gyre. *Proc. Natl. Acad. Sci. U.S.A.* **112**, 8008–8012 (2015).

44. C. O'brien, J. A. Peloquin, M. Vogt, M. Heinle, N. Gruber, P. Ajani, H. Andruleit, J. Arístegui, L. Beaufort, M. Estrada, D. Karentz, E. Kopczyńska, R. Lee, A. J. Poulton, T. Pritchard, C. Widdicombe, Global marine plankton functional type biomass distributions: Coccolithophores. *Earth Syst. Sci. Data* **5**, 259–276 (2013).

45. H. Akaike, Information theory and an extension of the maximum likelihood principle, in *Selected Papers of Hirotugu Akaike* (Springer, 1998), pp. 199–213.

46. R. Killick, P. Fearnhead, I. A. Eckley, Optimal detection of changepoints with a linear computational cost. *J. Am. Stat. Assoc.* **107**, 1590–1598 (2012).

47. G. Schwarz, Estimating the dimension of a model. *Ann. Stat.* **6**, 461–464 (1978).

48. E. T. Buitenhuis, W. K. W. Li, D. Vaulot, M. W. Lomas, M. R. Landry, F. Partensky, D. M. Karl, O. Ulloa, L. Campbell, S. Jacquet, F. Lantoine, F. Chavez, D. Macias, M. Gosselin, G. B. McManus , Picophytoplankton biomass distribution in the global ocean. *Earth Syst. Sci. Data* **4**, 37–46 (2012).

49. Y.-W. Luo, S. C. Doney, L. A. Anderson, M. Benavides, I. Berman-Frank, A. Bode, S. Bonnet, K. H. Boström, D. Böttjer, D. G. Capone, E. J. Carpenter, Y. L. Chen, M. J. Church, J. E. Dore, L. I.

Falcón, A. Fernández, R. A. Foster, K. Furuya, F. Gómez, K. Gundersen, A. M. Hynes, D. M. Karl, S. Kitajima, R. J. Langlois, J. LaRoche, R. M. Letelier, E. Marañón, D. J. McGillicuddy Jr., P. H. Moisander, C. M. Moore, B. Mouriño-Carballido, M. R. Mulholland, J. A. Needoba, K. M. Orcutt, A. J. Poulton, E. Rahav, P. Raimbault, A. P. Rees, L. Riemann, T. Shiozaki, A. Subramaniam, T. Tyrrell, K. A. Turk-Kubo, M. Varela, T. A. Villareal, E. A. Webb, A. E. White, J. Wu, J. P. Zehr , Database of diazotrophs in global ocean: Abundance, biomass and nitrogen fixation rates. *Earth Syst. Sci. Data* **4**, 47–73 (2012).

50. K. Leblanc, J. Arístegui, L. Armand, P. Assmy, B. Beker, A. Bode, E. Breton, V. Cornet, J. Gibson, M.-P. Gosselin, E. Kopczynska, H. Marshall, J. Peloquin, S. Piontkovski, A. J. Poulton, B. Quéguiner, R. Schiebel, R. Shipe, J. Stefels, M. A. van Leeuwe, M. Varela, C. Widdicombe, M. Yallop , A global diatom database—Abundance, biovolume and biomass in the world ocean. *Earth Syst. Sci. Data* **4**, 149–165 (2012).
